# Examining the relationship between risky sexual behavior and suicidal thoughts among unmarried adolescents in India

**DOI:** 10.1038/s41598-023-34975-2

**Published:** 2023-05-12

**Authors:** Chanda Maurya, T. Muhammad, Shriya Thakkar

**Affiliations:** 1 Department of Survey Research and Data Analytics, International Institute for Population Sciences, Mumbai, India; 2 Department of Family and Generations, International Institute for Population Sciences, Mumbai, India; 3 Department of Sociology, Louisiana State University, 26, Stubbs Hall, Baton Rouge, LA 70803 USA

**Keywords:** Health policy, Health services, Public health, Risk factors

## Abstract

Addressing the problem of suicidal thoughts in adolescents requires understanding the associated risk factors. Multiple studies have shown that risky sexual behavior affected the adolescents’ psychological health that leads to their suicidal thoughts, behaviors and attempts. This study aimed to identify the association between various risky sexual behaviours and suicidal thoughts among unmarried adolescents in India. We used data collected from 4221 unmarried adolescent boys and 5987 unmarried adolescent girls aged 10–19 years, from the two rounds of the Understanding the Lives of Adolescents and Young Adults (UDAYA) survey. Descriptive analysis was done to observe changes in the selected variables from wave-1 to wave-2. Random effect regression analysis was used to estimate the association of suicidal thoughts among unmarried adolescents with their risky sexual behaviours. The percentage of adolescent boys having suicidal thoughts increased from 1.35% in wave 1 to 2.19% in wave 2. Among adolescent girls, the percentage increased from 2.92% in wave 1 to 5.05% in wave 2. A proportion of 3.26% adolescent boys had more than one sexual partner during wave 1 whereas in wave 2, it rose to 8.71%, while in case of adolescent girls, the estimates only increased from 0.26% at wave 1 to 0.78% at wave 2. Nearly 4.55% boys and 1.37% girls had early sexual debut. Almost five percentage boys were sexually active at wave 1 whereas in wave 2, it rose to 13.56%, while among adolescent girls, the estimates decreased from 1.54% at wave 1 to 1.51% at wave 2. Contraceptive use increased over time among both adolescent boy and girls. Also, a large proportion of adolescent boys reported watching pornography (27.08% at waive 1 and 49.39% at wave 2) compared to adolescent girls (4.46% at wave 1 and 13.10% at wave 2). Adolescents who had more than one sexual partner [Coef: 0.04; p < 0.001], exposed to early sexual debut [Coef; 0.019; p < 0.01], sexually active [Coef: 0.058; p < 0.001] and reported watching pornography [Coef: 0.017; p < 0.001] were more likely to have suicidal thoughts. Adolescent boys and girls with risky sexual behaviors are likely to be at a higher risk of suicidal ideation, and thus, they should be treated with special care and attention by local healthcare practitioners.

## Introduction

Suicide remains an important public health concern, globally accounting for more than 700,000 deaths. Among adolescents, fourth leading cause of death is suicide^1^. Suicide does not just occur in developed countries, but is a global phenomenon in all regions of the world. In fact, over 77% of global suicides occurred in low- and middle-income countries in 2019^1^. Historically, suicide rate within the Indian context has been compared to that of Australia and the US, and the rising rates over the past few decades align with the global trend^2^. Between 1978 and 1990, the suicide rates in India increased by 41.3%, rising from 6.3 per 100,000 to 8.9 per 100,000. This corresponded to a compound annual growth rate of 4.1% over the decade 1980 to 1990^3^. While the period between 2003 and 2006 observed a mixed trend, this was further followed by a rising trend from 2006 to 2010^4^. Between 2009 and 10, the rate rose to 11.4 per 100,000, indicating a 5.9% increase in the number of suicides^4^. Recent trends have shown an increase in suicide rates from 9.9 per 100,000 in 2017 to 10.2 in 2018, with adolescents accounting for the high-risk group for suicidal ideation^5,6^. Additionally, there was a gender disparity in suicide rates with 14.3 and 20.3 per 100,000 of male and female adolescents, respectively, commintting suicide^7^. Globally, according to a school-based survey of youth aged 13–17 years, the 12-month pooled prevalence of suicidal ideation among males and females were reported to be 16.2% and 12.2% respectively. The prevalence of suicidal ideation with a specific plan was 8.3% for females and 5.8% for males^8^.

Suicide related thoughts and behaviours among adolescents is a serious public health problem, however, with the availability of low cost interventions, suicides are preventable with timely response^1^. Considering the critical importance of suicide prevention, a large body of literature has investigated the influence of protective factors against suicidal thoughts and behaviours (STB) during adolescence^9^. However, there is dearth of studies on risk factors associated with the suicidal thoughts among adolescents, especially in resource-limited settings.

Risk factors for suicide having many aspects to be considered, and include harmful use of alcohol, smoking^10^, abuse in childhood^11^, depression or hopelessness^12,13^, impulsive behavior and physical activity^14,15^, stigma against help-seeking, barriers to accessing care and increased access to means of suicide^16–18^. Similarly, digital media, like any other media, can play a significant role in either enhancing or weakening suicide prevention efforts^19^. Most of the studies on the risk factors of adolescent suicide in India focused on the family issues such as lack of parental emotional support and poor parent–child communication; school-related factors such as school dropout, academic failure, harassing or abusive school environment^20–22^. Several studies were also conducted on the risk factors of suicidal thoughts including alcohol or tobacco use, aggressive behavior, stress and optimism, self-harm, body image consciousness, depressive symptoms, physical, sexual and emotional abuse, independent decision making, etc.^23–25^. On the other hand, multiple studies across the globe have shown that risky sexual behavior affected the adolescents’ psychological health that leads to suicidal thoughts, behaviors and attempts^26–28^.

Risky sexual behaviour can be explained as any sexual activity that increases the risk of contracting HIV or other STI or becoming pregnant^29,30^. Risky sexual behaviour among adolescents includes early sexual debut, unprotected sexual activity, inconsistent use of condoms, high-risk partners (e.g. injection drug users, survival sex [sex in exchange for money, drugs, food, or shelter]), or sex with more than one partners or with partner who has other partners or more than one partner at a time^31,32^. Risky sexual behaviour is also an important aspect related to sexuality which increases the susceptibility of an individual to his/her problems related to sexuality and reproductive health, unwanted and unplanned pregnancy, abortion, and psychological distress^33–35^. Numerous studies have been conducted on adolescents’ suicidal thoughts and only few have properly addressed its linkage with risky sexual behaviour. In particular, no study has been carried out in order to identify the relationship between specific sexual behaviours and suicidal thoughts among adolescents in Indian context. Some studies conducted in similar settings have reported an association between adolescent sexual behaviour and suicidal ideation^36,37^. Therefore, the aim of this study to identify the association between various risky sexual behaviours and suicidal thoughts among unmarried adolescent boys and girls using the large scale survey data from the two least developed states (Uttar Pradesh and Bihar) in India.

## Methods

### Data

This study used two-wave data from Understanding the lives of adolescents and young adults (UDAYA) project survey^31^. The first wave of the survey was conducted in two states namely, Uttar Pradesh and Bihar in 2016 in India. The UDAYA collected detailed information on media, family, assets acquired in adolescence, community environment, and quality of transitions to young adulthood indicators. The sample size of the suyvey in wave 1 for both Uttar Pradesh and Bihar was 10,350 adolescents (aged 10–19 years). The required sample for each sub-class of adolescents was determined at 920 younger and 2350 older boys, 630 younger and 3750 older girls, and 2700 married girls^31^. The UDAYA adopted a multistage systematic sampling design to provide the estimates for states and also for the urban and rural areas. More details about the study design and sampling procedure have been published elsewhere^31^. At both the waves, written consent was obtained from the respondents. Finally, in wave 1, with a response rate of 92%, almost 10,000 adolescents were interviewed using the structured questionnaire from both Uttar Pradesh and Bihar.

**Figure 1:**
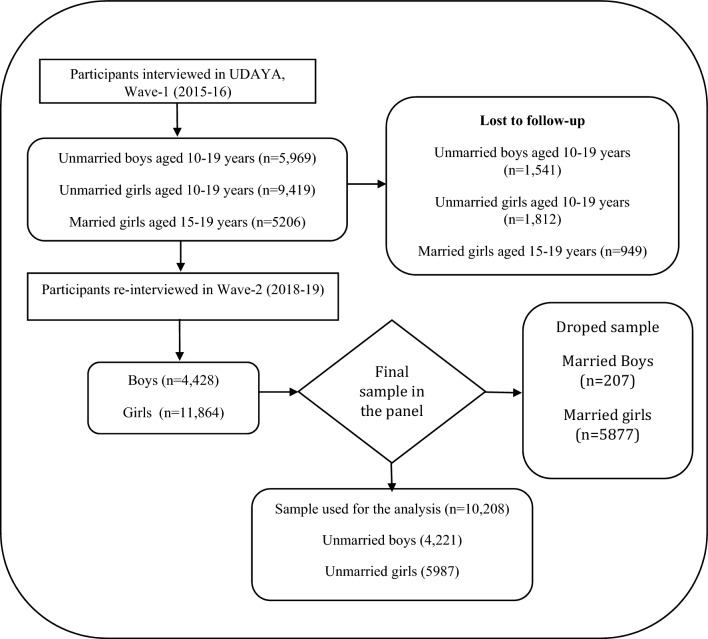
Flow chart depicting the selection of study participants.

Moreover, during wave-2 (2018–2019), the survey collected information from the participants who were successfully interviewed in 2015–2016 and who consented to be re-interviewed. Of those who were eligible for the re-interview, the survey re-interviewed 4567 boys and 12,251 girls (married and unmarried). After excluding the respondents who gave an inconsistent response to age and education at the follow-up survey (3%), the final follow-up sample covered 4428 boys and 11,864 girls^31^. The effective sample size for the present study was 4,221 unmarried adolescent boys and 5987 unmarried adolescent girls aged 10–19 years at wave 1 and wave 2. For the strongly balanced dataset, cases whose follow-up was lost were excluded from the sample. For the longitudinal analysis, data were set by using the *xtset* command in STATA 15. Figure 1 presents the process of selecting the sub-sample for conducting this research.

### Outcome variable

Suicidal thought was the outcome variable in this study. Suicidal thought was measured by the following question: “Have you ever seriously considered attempting suicide during the past 12 months?” The respondents answered as “yes” or “no”.

### Exposure variables

Based on the previous literature, five risky sexual behaviors were selected for this study. (1) More than one sexual partner^32^: the question on the number of sexual partners was: “In all, how many boyfriends have you had in the last 3 years?” and was recoded as “one or no” and “more than one”. (2) The early sexual debut: it was created using the question on age at first sex among unmarried adolescents; that is, adolescents who experienced their first sexual intercourse before 18 years of age were coded as 1 representing early sexual debut and 0 otherwise^32^. (3) Sexually active: it was recoded as “yes” and “no”. (4) Contraceptive use: it was recoded as “yes” and “no”^32^. (5) Watching pornography: it was recoded as “yes” and “no”.

Other covariates of the study included the following. Age group was recoded as “10–14 years” and “15–19 years”. Sex was recoded as “male” and “female”. Years of education was categorized as “no”, “upto 10 years” and “more than 10 years”. Paid work was recoded as “no” and “yes”. Peer connection was categorized as “good” having 5 or more friends and “bad” having four and less friends. Mass media exposure was recoded as “no” and “yes”. Media exposure contains exposure to newspaper, television and radio. Internet access was recoded as “yes” and “no”. Depressive symptoms were assessed by using nine questions; the respondent was asked about the symptoms for the past two weeks only before survey. The questions included, (i) had trouble falling asleep or sleeping too much, (ii) feeling tired or having little energy, (iii) poor appetite or eating too much, (iv) trouble concentrating on things, (v) had not much pleasure or interest in doing things, (vi) feeling down, depressed, or hopeless, (vii) feeling bad about yourself, (viii) been moving or speaking slowly, (ix) had thoughts about respondent would be better off dead. All the above questions measured asked on a likert scale of four, i.e. 0 “not at all,” 1 “less than once in a week,” 2 “one week or more” and 3 “nearly every day.” Variable was then generated using the egen command in STATA 14 at scale of 27 points^33^. The variable was recorded as 1 “Yes” having depressive symptoms and 0 “No” not having depression symptoms^33^. Substances use was recoded was “yes” ever use smokeless tobacco, smoking or drinking and “no” otherwise. Mother’s education was recoded as “illiterate” and “literate”. Caste was categorized as SC/ST and non-SC/ST. Religion was categorized as Hindu and Non-Hindu. Wealth index was categorized as poorest, poorer, middle, richer and richest. Place of residence was categorized as urban areas and rural areas. States were categorized as Uttar Pradesh and Bihar.

### Statistical analysis

Descriptive analysis was used to report the characteristics of unmarried adolescent boys and girls at wave-1 (2015–2016). The changes in selected variables were observed from wave-1 to wave-2 (2018–2019). Moreover, random effect regression analysis was used to estimate the association of change in suicidal thoughts among unmarried adolescents with individual risky sexual behaviours. The Random Effects regression model has a specific benefit. it is used to estimate the effect of individual-specific that are inherently unmeasurable e.g., mother’s education, caste, religion, wealth status, place of residence, parental co-residence, states. Such individual-specific effects are often encountered in panel data studies. For the best fit model, Hausman test was performed to differentiate between fixed effect and random effect^38^. The Hausman test revealed that random effect model was the best model for the analysis.

### Ethics approval

The data is freely available in the public domain on request and the study has been approved by the Population Council Review Board, New Delhi. All methods were performed following the relevant guidelines and regulations.

### Consent to participate

Informed consent has been taken from the participants in verbal and written forms.

## Results

The socio-economic profile of unmarried adolescent boys and girls are presented in Table 1. The estimates are from the baseline data set and it was assumed that none of the charactistics change over time among adolescent boys and girls.

**Table 1:** Socio-economic characteristics of study population, 2015–2016.

Background charactistics	Male		Female	
	N	%	N	%
Mother’s education				
No	2711	69.23	3725	66.30
Yes	1510	30.77	2262	33.70
Parental co-residence				
No	121	2.32	195	2.69
With one parent	619	14.65	891	14.46
With both parent	3481	83.03	4901	82.85
Religion				
Hindu	3537	84.42	4390	75.79
Non-Hindu	684	15.58	1597	24.21
Caste				
SC/ST	1012	26.44	1203	22.81
Non SC/ST	3209	73.56	4784	77.19
Wealth index				
Poorest	391	11.30	492	10.89
Poorer	641	19.10	748	15.68
Middle	862	22.39	1106	20.48
Richer	1142	23.85	1702	25.71
Richest	1185	23.37	1939	27.24
Place of residence				
Urban	1933	17.39	2901	19.44
Rural	2288	82.61	3086	80.56
State				
Uttar Pradesh	2185	67.83	3476	75.61
Bihar	2036	32.17	2511	24.39
Total	4221	100.00	5987	100.00

*SC/ST* scheduled caste/scheduled tribe.

**Figure2:**
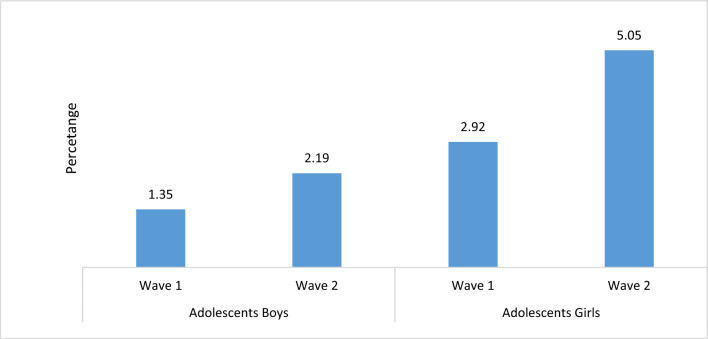
The Percentage of adolescents boys and girls having suicidal thoughts, wave 1 (2015–16), wave 2 (2018–19).

Figure 2 presents the changes in the suicidal thoughts among adolescent boys and girls. The percentage of adolescent boys who had suicidal thoughts increased from 1.35% at wave 1 to 2.19% at wave 2 while among adolescent girls, the percentage increased from 2.92% at wave 1 to 5.05% at wave 2.

The characteristics of the participants of UDAYA wave 1 and wave 2 are presented in Table 2. A proportion of 3.26% adolescent boys had more than one sexual partner during wave 1 whereas in wave 2, it rose to 8.71%, while in case of adolescent girls, the estimates only increased from 0.26% at wave 1 to 0.78% at wave 2. There was significant difference in reporting early sexual debut among adolescent boys and girls, nearly 4.55% boys and 1.37% girls had early sexual debut. Almost five percentage of adolescent boys were sexually active at wave 1 whereas, in wave 2, it rose to 13.56%, while among adolescent girls, the estimates decreased from 1.54% at wave 1 to 1.51% at wave 2. Contraceptive use increased over time among both adolescent boy and girls. Also, a large proportion of adolescent boys reported watching pornography (27.08% at waive 1 and 49.39% at wave 2) compared to adolescent girls (4.46% at wave 1 and 13.10% at wave 2).

**Table 2:** Summary statistics for explanatory variables used in the analysis of UDAYA wave-1 and wave-2.

Variables	Adolescent boys		Adolescent girls	
	Wave 1	Wave 2	Wave 1	Wave 2
More than one sexual partner				
No	96.74	91.29	99.74	99.22
Yes	3.26	8.71	0.26	0.78
Early sexual debut				
No	95.45	95.45	98.63	98.63
Yes	4.55	4.55	1.37	1.37
Sexually active				
No	95.10	86.44	98.46	98.49
Yes	4.90	13.56	1.54	1.51
Contraceptive use				
No	98.07	94.67	99.77	99.25
Yes	1.93	5.33	0.23	0.75
Watching pornography				
No	72.92	50.61	95.54	86.90
Yes	27.08	49.39	4.46	13.10
Age group (in years)				
10–14	40.89	41.53	23.39	24.83
15–19	59.11	58.47	76.61	75.17
Years of education				
No	2.3	2.3	4.82	4.82
Up to 10 years	81.72	59.28	71.05	46.93
More than 10 years	15.98	38.42	24.13	48.25
Paid work				
Yes	22.25	41.89	17.03	29.71
No	77.75	58.11	82.97	70.29
Peer connection				
Bad	68.54	47.85	71.87	63.89
Good	31.46	52.15	28.13	36.11
Mass media exposure				
No	2.52	1.86	8.54	6.65
Yes	97.48	98.14	91.46	93.35
Internet access				
No	72.24	25.88	91.48	60.18
Yes	27.76	74.12	8.52	39.82
Depression				
No	92.61	83.48	86.94	75.17
Yes	7.39	16.52	13.06	24.83
Substance use				
No	87.24	70.71	98.68	96.71
Yes	12.76	29.29	1.32	3.29

Further, about one fifth of the adolescent boys were engaged in paid work at wave 1 whereas, it increased up to 42% at wave 2, while among adolescent girls, the estimates increased from 17.03% at wave 1 to 29.71% at wave 2. The exposure to mass media except techno-media was almost universal for adolescent boys while among adolescent girls, it increased from 91.46% at wave 1 to 93.35%. There was a sharp rise in the internet use among both adolescent boys and girls. Nearly 27.76% adolescent boys used internet in wave 1 whereas in wave 2, there was sharp rise to 74.12%, while among adolescent girls it increased from 8.52% at wave1 to 40% wave 2. About 7.39% adolescent boys had depressive symptoms in wave 1 whereas in wave 2, there were 16.52% adolescent boys who had depressive symptoms, while among adolescent girls, the estimates increased from 13.06% at wave 1 to 24.83% at wave 2. Substances use among adolescent boys increased from 12.76% at wave 1 to 29.29% at wave 2, while among adolescent girls, it increased from 1.32% at wave 1 to 3.29% at wave 2.

The bivariate associations between explanatory variables and outcome variable are shown in Table 3. The prevalence of suicidal thoughts was higher among those having more than one sexual partner (Wave 1: 12.3% and Wave 2: 12.6%), having early sexual debut (Wave 1: 12.4% and Wave 2: 7.5%), being sexually active (Wave 1: 13.8% and Wave 2: 10.1%), using contraceptives (Wave 1: 11.9% and Wave 2: 11.6%), and watching pornography (Wave 1: 4.2% and Wave 2: 5.3%). Adoelscents aged 15–19 at wave 1 were having higher prevalence of suicidal thoughts at both the waves. Females and depressed adolescents were having higher prevalence of suicidal thoughts.

**Table 3:** Association of explanatory variables to the suicidal thoughts among adolescents and young adults, UDAYA, wave-1 and wave-2.

Variables	Suicidal thoughts			
	Wave 1	P-value	Wave 2	P-value
More than one sexual partner				
No	2.1	< 0.001	3.5	< 0.001
Yes	12.3		12.6	
Early sexual debut				
No	2.0	< 0.001	3.8	< 0.001
Yes	12.4		7.5	
Sexually active				
No	1.9	< 0.001	3.4	< 0.001
Yes	13.8		10.1	
Contracptive use				
No	2.2	< 0.001	3.6	< 0.001
Yes	11.9		11.6	
Watching pornography				
No	2.0	< 0.001	3.3	< 0.001
Yes	4.2		5.2	
Age group (in years)				
10–14	0.3	< 0.001	2.3	< 0.001
15–19	3.1		4.6	
Sex				
Male	1.3	< 0.001	2.2	< 0.001
Female	2.9		5	
Years of education				
No	2.1	< 0.001	6.5	0.192
Upto 10 years	1.9		3.9	
More than 10 years	3.5		3.6	
Paid work				
Yes	3.8	< 0.001	5	< 0.001
No	1.9		3.2	
Peer connection				
Bad	2.2	0.206	3.4	0.04
Good	2.4		4.5	
Mass media exposure				
No	1.6	0.664	2.6	0.229
Yes	2.3		3.9	
Internet access				
No	2.0	0.001	3.7	0.376
Yes	3.7		4	
Depression				
No	1.1	< 0.001	1.7	< 0.001
Yes	11.7		11.9	
Substance use				
No	2.1	< 0.001	3.8	0.159
Yes	4.7		4.3	
Parental co-residence				
No	2.1	0.528	3.8	0.009
With one parent	2.2		5	
With both parents	2.3		3.7	
Mother's education				
Illiterate	2.2	0.287	3.8	0.648
Literate	2.4		4.1	
Caste				
Non SC/ST	3.1	0.537	4.7	0.003
SC/ST	2.0		3.6	
Religion				
Hindu	2.2	0.198	3.9	0.363
Non Hindu	2.3		3.6	
Wealth index				
Poorest	1.6	0.126	3.8	0.117
Poorer	1.9		3.6	
Middle	2.3		4.4	
Richer	2.4		4.1	
Richest	2.7		3.4	
Place of residence				
Rural	2.9	0.007	4.8	< 0.001
Urban	2.1		3.7	
State				
Uttar Pradesh	2.6	0.002	3.7	0.137
Bihar	1.5		4.3	
Total	2.3		3.9	

Table 4 represents the estimates from random effect of suicidal thoughts among adolescents. It was found that adolescents [Coef: 0.04; p < 0.001] who had more than one sexual partner were more likely to have suicidal thought in comparison to those who had no sexual partner or had only one sexual partner. Adolescents who reported early sexual debut were positively associated with suicidal thoughts compared to those who did not report early sexual debut [Coef; 0.019; p < 0.01]. Sexually active adolescents were more likely to have suicidal thoughts [Coef: 0.058; p < 0.001] than those who were sexually inactive. Adolescents who used contraceptives were negatively associated with the suicidal thoughts [Coef: -0.009; p > 0.01] in comparison to those who had not used contraceptives. Adolescents who reported watching pornography were more likely to have suicidal thoughts [Coef: 0.017; p < 0.001] as compared to those who did not report watching pornography. It was further found that older adolescents were more likely to have thoughts about suicide than younger adolescents. Adolescent girls [Coef: 0.033; p < 0.001] were more likely to have suicidal thoughts in comparison to adolescent boys. Adolescents who had not done paid work were more likely to have suicidal thoughts than their counterparts. Adolescents who suffered from depressive symptoms [Coef: 0.13; p < 0.001] were positively associated with having thoughts about suicide. Adolescents from the urban place of residence [Coef: 0.009; p = 0.001] were more likely to have suicidal thoughts in reference to those from the rural place of residence.

**Table 4:** Estimated effects of explanatory variables on suicidal thought from random effect models.

Variables	Random effect coefficient (CI)
More than one sexual partner	
No(Ref)	
Yes	0.04***(0.02–0.06)
Early sexual debut	
No(Ref)	
Yes	0.019*(0–0.04)
Sexually active	
No(Ref)	
Yes	0.058***(0.04–0.08)
Contraceptive use	
No(Ref)	
Yes	− 0.009 (− 0.03–0.02)
Watching pornography	
No(Ref)	
Yes	0.017***(0.01–0.02)
Age group (in years)	
10–14(Ref)	
15–19	0.011***(0–0.02)
Sex	
Male (Ref)	
Female	0.033***(0.03–0.04)
Years of education	
No (Ref)	
Up to 10 years	0.014*(0–0.03)
More than 10 years	0.015***(0.01–0.02)
Paid work	
No (Ref)	0.009***(0–0.02)
Yes	
Peer connection	
Bad(Ref)	
Good	0.002 (0–0.01)
Mass media exposure	
No (Ref)	
Yes	0.007 (− 0.01–0.02)
Internet access	
No (Ref)	
Yes	0.008**(0–0.01)
Depression	
No	
Yes	0.13***(0.12–0.14)
Substance use	
No(Ref)	
Yes	− 0.001 (− 0.01–0.01)
Parental co-residence	
No (Ref)	
With one parent	0.011 (0–0.03)
With both parents	0.002 (− 0.01–0.02)
Mother’s education	
Illiterate (Ref)	
Literate	− 0.004 (− 0.01–0)
Caste	
Non SC/ST (Ref)	
SC/ST	0.005 (0–0.01)
Religion	
Hindu (Ref)	
Non Hindu	− 0.005 (− 0.01–0)
Wealth index	
Poorest (Ref)	
Poorer	− 0.001 (− 0.01–0.01)
Middle	0.007 (0–0.02)
Richer	0.002 (− 0.01–0.01)
Richest	− 0.002 (− 0.01–0.01)
Place of residence	
Rural (Ref)	
Urban	0.009***(0–0.02)
State	
Uttar Pradesh(Ref)	
Bihar	0 (− 0.01–0)
Years	
2014–15(Ref)	
2018–19	0.01***(0–0.02)
Sigma_u	0.033
Sigma_e	0.175
Rho	0.035

*Ref* Reference, *CI* confidence interval, *SC/ST* scheduled caste/scheduled tribe.

**if p < 0.05.

***if p < 0.001.

## Discussion

A number of empirical studies have been undertaken to explore the prevalence of depressive symptoms among young adults^39,40^; however, fewer studies have examined the increased risks associated with sexual health behaviour among adolescents. Further, research within the broader domain of mental health and suicidal ideation within younger adults (especially women) remain moderately sparse^41^. Thus, through a rich synthesis of the analyses, our present study explores the association of risky sexual health behaviour and suicidal ideation among adolescents and attempts to fill the gap within the relevant literature. In our results, we observed that suicidal ideation was positively correlated with a higher number of sexual partners, i.e., adolescents who had a higher number of sexual partners were more prone to frequent suicidal thoughts as opposed to their immediate counterparts. Similar results have been noted in previous studies that found individuals with inconsistent use of protection and higher sexual partners were twice as likely to suffer from suicidal thoughts^42^. Further, the results were consistent with findings from other underdeveloped nations such as, Malawi, indicating that suicidal planning and ideation was clearly higher among school-attending adolescents with early sexual debut among other factors^43,44^. However, research suggests that good parental communication and higher levels of parental monitoring are keys to preventing suicidal ideation among adolescents^45^. In order for parents to address such a sensitive topic with their adolescent children, it is important to provide awareness to parents with accurate information on areas surrounding adolescent sexuality. This could lead to effective methods for initiation of discussions relating to adolescent sexuality between parents and adolescents at home, as well as management strategies to tackle anxieties and concerns around risky sexual behaviors among adolescents.

Further, a conspicuous gendered division suggested that adolescent boys were more sexually active between waves 1 and 2 with increased early sexual debut as compared to adolescent girls. Previously, gender specific research studies have attributed early age of sexual debut primarily with higher suicidal ideation and females were more likely to exhibit tendencies of suicidal thought whereas males had a higher prediction of suicidal rates^46,47^. Similar findings were further substantiated with other studies where adolescent girls were found to be prone to suicidal tendencies as compared to adolescent boys in the Indian^5^ as well as German context^48^. Studies have attributed this gendered division to higher depressive symptoms among adolescent during the post-pubertal period^49^. Further, to investigate the gendered division in the area of early sexual debut among Indian adolescents, one of our studies recently found that early sexual debut is commonplace among adolescent boys who have a reported family history of substance users^50^. Earlier, research has also found that individuals who indicated severity and frequency of maltreatment were often more prone to violent behavior, early sexual debut, and substance abuse^51–53^. This could be supported by the cumulative risk theory which advances the argument that the impact of adverse events on an individual’s life is directly proportional to the number of exposures^52^. This directly translates to the understanding that the experience of multiple adverse events will lead to worse individual outcomes as opposed to a single event exposure^54,55^.

Another important finding suggests that adolescent boys were, on an average, more sexually active than adolescent girls, indicating a sharp increase in sexual activity between waves 1 and 2. Similar results have been reported in earlier studies showing increased sexual activity among adolescent boys is marked by early sexual debut^56,57^. Previous scholars have ascribed this striking gendered division to shame, guilt, and more negative attitude towards sex among females compared to males^56^. Additionally, females are more culturally sensitive to their sexual health within most Asian countries that stave off them from engaging in sexual activities during their pubertal growth^58^. However, we found that adolescent girls, on average, engaged more in unprotected sex than adolescent boys across both waves. Adolescent boys, on the other hand, indicated a sharp increase in contraceptive usage between waves 1 and 2. Previous research has corroborated similar results and observed a low uptake of contraception among adolescent girls and women across and low- and middle-income countries^59^. Poor knowledge of contraception, limited access to social and financial resources, weaker bargaining power, and, ill exposure to perpetuating myths and misinformation are some of the factors that have contributed to the negligence of contraceptive use among adolescent girls^60,61^. Additionally, girls within developed and developing countries are subjected to the risk of stigma around casual sex that further, limit their usage of contraception^62^.

Our analyses also reported that adolescent girls, on average, were highly prone to depression across both waves as compared to adolescent males. Further, the reported figures roughly doubled from wave 1 (13.06) to wave 2 (24.83) for adolescent girls in the context of India. Several background characteristics also explicate the onset of depression as a result of risky sexual behaviour among adolescents. For instance, adolescent girls, on average, had lower education across all categories as compared to adolescent boys between waves 1 and 2. According to previous studies, higher education among adolescent girls contribute to better mental health enhancing their personal skills and employability, which further reduces the risk of depression and suicidal ideation^63^. Consistent with our findings, previous studies reported that adolescent girls are almost twice as likely to experience depression as compared to their male counterparts^64^. Adolescence is observed as a tender age which marks a crucial transition between childhood and adulthood, characterized by multiple physiological changes among females. Previous research has attributed higher sensitivity and neurobiological vulnerability among adolescent girls reflecting their inability to cope up with the marked changes, which, in turn, increases the risk of depression as well as suicidal ideation^65^.

### Strength and limitations of the study

The study findings should be explained in light of some limitations and strengths. First, considering the sensitivity of the subject, there could be a higher possibility of severe under-reporting of information on suicidal ideation. Further, information gathered on depression was self-reported and was not subjected to any clinical diagnosis which could lead to issues of reliability. Since the nature of data was largely cross-sectional, it limits our capacity in drawing causal inference. Finally, since the study was conducted in two states of Northern India, namely, Bihar and Uttar Pradesh, it raises issues of generalizability to the wider population within the Indian context. However, despite the given limitations, our study is among the first of its kind to examine the association of risky sexual behaviour and suicidal ideation among the adolescents in the states of Bihar and Uttar Pradesh. Research suggests that adolescent age groups are one the most overlooked group of patients when it comes to clinical health and emotional well-being^66^. Thus, through the present study, we attempt to recentre the focus on the emotional needs and psychological well-being of adolescents, especially girls.

## Conclusions

Adolescent boys and girls with risky sexual behavior are likely to be at a higher risk of suicidal ideation, and thus, they should be treated to special care and attention by local healthcare practitioners. The observed gendered division in sexual behaviour underscores the need for gender specific guidelines and solutions to achieve better sexual and reproductive outcomes among the adolescents. Further, we propose effective policy interventions through youth-accessible mass media (e.g. radio) aimed at increasing awareness of sexual health and contraceptive use among adolescent boys and girls. Family support programs should incorporate comprehensive sex education, with a focus on reproductive health, as a universal component to promote a shift towards fostering a completely healthy adolescent lifestyle. In addition, to effectively educate adolescents about reproductive health, it would be beneficial if health workers impart sex education awareness to both parents and teachers in a formal school setting. This could involve conducting workshops for teachers which would enable them to cover sensitive aspects of reproductive health with students, as well as, workshops for parents and children to improve family communication thereby extending guidance on how to effectively navigate various issues related to sexual behavior.

## Data Availability

The datasets generated and analysed during the current study are available in the GIRL Center Dataverse (Population Council) repository and accessible on reasonable request through following the link, https://dataverse.harvard.edu/dataset.xhtml?persistentId=doi:10.7910/DVN/RRXQNT.
